# Hypoalbuminemia and Nutritional Status in Docetaxel-Induced Erythema Multiforme: A Case Report

**DOI:** 10.7759/cureus.95618

**Published:** 2025-10-28

**Authors:** Aisha Nadeem, Abel Zachariah, Rakesh Mehra

**Affiliations:** 1 Clinical Oncology, New Cross Hospital, Birmingham, GBR; 2 Oncology, New Cross Hospital, Birmingham, GBR

**Keywords:** docetaxel, drug toxicities, erythema multiforme, erythema multiforme major, hypoalbuminemia, phesgo

## Abstract

Docetaxel is a widely used chemotherapy agent used in breast cancer. Although uncommon, docetaxel-induced severe cutaneous adverse reactions including erythema multiforme major, Stevens-Johnson syndrome and toxic epidermal necrolysis have been reported. We present a case of a patient with breast cancer developing an erythematous rash covering 20% of her total body surface area where malnutrition and severe hypoalbuminemia of 17g/L were present. This occurred 17 days post cycle five administration of her chemotherapy - docetaxel and Phesgo (pertuzumab, trastuzumab and hyaluronidase). Punch biopsy confirmed erythema multiforme-type cutaneous reaction. Despite appropriate medical management, the patient passed away 10 days after hospital admission due to cancer progression. The pharmacokinetics of docetaxel are discussed, and we conclude that due to its extensive protein binding, hypoalbuminemia and malnutrition may contribute to the risk of docetaxel-induced toxicities. This case highlights the potential need to monitor albumin and nutritional status in patients receiving docetaxel to mitigate toxicities.

## Introduction

Erythema multiforme is a hypersensitivity reaction causing erythematous lesions to evolve on the body with mucosal involvement. It can be caused by infections including herpes simplex and cytomegalovirus and various drugs such as antibiotics, anti-epileptics, and non-steroidal anti-inflammatory drugs [1].

Docetaxel is a widely used taxane chemotherapy agent, frequently used in the treatment of breast cancer and non-small cell lung cancer. It has been commonly associated with dermatological reactions including alopecia, nail dystrophy and hand-foot syndrome [2]. Severe cutaneous adverse reactions (SCARs) to docetaxel, including erythema multiforme, Stevens-Johnson syndrome (SJS) and toxic epidermal necrolysis (TEN), are extremely uncommon and have rarely been reported in the literature [3-7]. 

Docetaxel is extensively protein bound, with approximately 94-97% of docetaxel being bound to predominantly alpha-one-acid glycoprotein (AAG) and secondarily to albumin [8]. Therefore, hypoalbuminemia may lead to increased unbound docetaxel levels. Hypoalbuminemia and malnutrition are common findings in patients with advanced cancer [9]; however, their role in modulating docetaxel-induced skin toxicities has not been well studied. The current Clatterbridge systemic anti-cancer therapy (SACT) protocol provides dose adjustments and recommendations for docetaxel in renal and hepatic impairment but does not mention albumin levels in its guidance [10]. Given the rarity of SCARs with docetaxel use and the lack of guidance on hypoalbuminemia, we present a case of docetaxel-induced erythema multiforme in a patient with low albumin levels. To our knowledge, this is the first case report that presents a possible correlation between hypoalbuminemia and cutaneous toxicities, based on the pharmacokinetics of docetaxel.

## Case presentation

A 62-year-old woman with HER2-positive breast cancer with metastases to the liver, lungs and bone, was receiving palliative chemotherapy with docetaxel and Phesgo - a combination of pertuzumab, trastuzumab and hyaluronidase. Prior to treatment initiation, she had a performance status of one. After cycle two, she developed a grade two skin rash on the hands and face and grade two mucositis. She was treated as an outpatient with emollients including Cetraben and hydrocortisone - her docetaxel dose was then reduced to 75% for subsequent cycles (Table 1). She continued to suffer from mucositis in the following cycles, with the most severe presentation after cycle four. 

**Table 1:** Docetaxel and Phesgo Dosing Schedule and Modifications This table presents a summary of all administered cycles of docetaxel and Phesgo including treatment dates, doses and the clinical rationale for dose modifications.

Cycle Number	Date of Cycle	Phesgo Dose (mg)	Reason for Phesgo Dose	Planned Docetaxel Dose as per Protocol (mg/m2)	Planned Dose Modification (%)	Body Surface Area (m^2^)	Actual Docetaxel Dose (mg)	Cumulative Dose (mg)	Reason for Docetaxel Dose
1	21/05/2025	1800	Standard loading dose for cycle 1 as per hospital protocol	75	0	1.92	148	148	75mg/m^2^ given as standard loading dose as per hospital trust protocol for cycle 1
2	11/06/2025	1200	Standard dose for cycle 2 onwards as per hospital protocol	100	90	1.99	180	328	100mg/m^2^ given as standard dose for patients with performance status 0-1 as per hospital trust protocol for cycle 2 onwards. Dose reduced to 90% due to patient being admitted for neutropenia 5 days after cycle 1
3	10/07/2025	1200	Standard dose for cycle 2 onwards as per hospital protocol	100	75	1.90	148	476	Dose reduced to 75% due to grade 2 mucositis and skin rash
4	07/08/2025	1200	Standard dose for cycle 2 onwards as per hospital protocol	100	75	1.86	148	624	Dose remains at 75% due to previous toxicities
5	28/08/2025	1200	Standard dose for cycle 2 onwards as per hospital protocol	100	75	1.83	132	756	Dose remains at 75% due to previous toxicities

**Figure 1:**
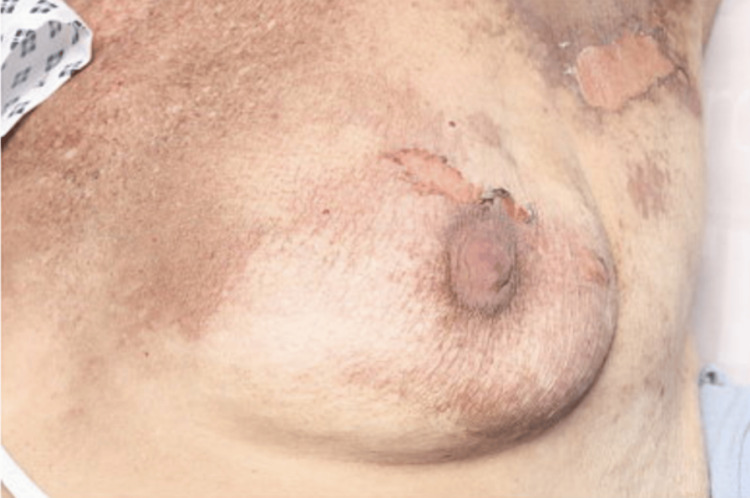
Clinical Photograph of the Left Breast Well demarcated, erythematous lesions with superficial peeling of the skin, resembling a burn-like appearance, consistent with docetaxel-induced erythema multiforme.

**Figure 2:**
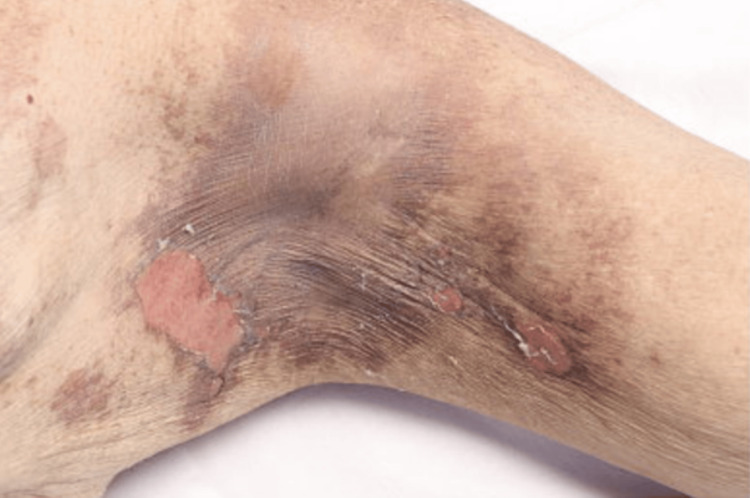
Clinical Photograph of the Left Axilla Clinical photograph showing multiple erythematous lesions at the left axilla with desquamation of the skin, representing docetaxel-induced erythema multiforme.

Seventeen days after cycle five administration, she presented with peeling, erythematous, burn-like lesions covering 20% of her body surface area (Figures 1, 2). The skin involved included the cheeks, mouth, neck, axillae, dorsum of hands, groin, and buttocks alongside mucositis on the eyelids and lips. The working diagnosis by the dermatology team was suspected SJS or staphylococcal scalded skin syndrome secondary to docetaxel usage. The Severity of Illness Score for Toxic Epidermal Necrolysis (SCORTEN) was calculated as two. Blood results showed moderate hypoalbuminemia of 23g/L two days prior to chemotherapy infusion, and this deteriorated to 17g/L at presentation. She also had raised inflammatory markers with normal kidney and liver function (Table 2).

**Table 2:** Laboratory Test Results Laboratory results showing reducing albumin levels as treatment progresses leading to severely low albumin on presentation with erythema multiforme.

Date	05/06/2025 One day prior to cycle two	26/08/2025 Two days prior to cycle five	14/09/2025 Day one of Admission	16/09/2025 Day three of Admission	23/09/2025 Day 10 of Admission (Last bloods)	Reference Ranges
Sodium	138	132	136	129	140	133-146mmol/L
Potassium	4.8	3.4	3.8	4.4	2.8	3.5-5.3mmol/L
Urea	2.4	2.1	6.1	9.7	5.9	2.5-7.8mmol/L
Creatinine	49	53	51	70	42	50-98umol/L
eGFR	>90	>90	>90	80	>90	>59mL/min/1.73m^2^
C-Reactive Protein (CRP)	Not recorded	Not recorded	206	107	54	0.0-5.0mg/L
Bilirubin	15	22	30	19	21	5-26umol/L
Alanine Aminotransferase (ALT)	45	18	32	28	29	0-55IU/L
Albumin	24	23	17	12	17	30-45g/L
Alkaline Phosphatase (ALP)	639	125	191	172	172	30-130IU/L
Adjusted Calcium	2.33	2.4	Not recorded	2.43	2.24	2.20-2.60mmol/L
Magnesium	Not recorded	0.69	Not recorded	Not recorded	0.71	0.7-1.0 mmol/L
Phosphate	Not recorded	Not recorded	Not recorded	Not recorded	0.85	0.8-1.5 mmol/L
Haemoglobin	133	130	137	127	123	115-165 g/L
White blood cells	9.1	11.7	28.8	17.9	21.8	4.0-11.0 x10^9^/L
Platelets	281	330	144	132	129	150-450 x10^9^/L

Intensive care admission was considered however after a multidisciplinary discussion, it was decided that the patient would not be a suitable candidate for admission due to her poor functional baseline prior to admission. Intravenous piperacillin/tazobactam was started due to markedly elevated inflammatory markers and suspicion of a superimposed infection. Dermatology review was sought, and they advised supportive management including intravenous fluids, regular emollient usage every two hours, fusidic acid/betamethasone valerate cream twice a day, non-adherent dressing application and pressure sore prevention. She was also started on intravenous methylprednisolone 1mg/kg for three days which dermatology advised stopping due to risk of immunosuppression with suspected sepsis. Her mucositis was treated with benzydamine and nystatin, both given four times a day. 

Dietician input was sought as the patient reported poor nutrition secondary to reduced appetite and mucositis. They noted she had weight loss of 15.3% over four weeks increasing her risk of malnutrition. Oral nutritional supplements were prescribed; however, maintaining optimal nutrition levels remained difficult due to her mucositis. Nasogastric feeding was not attempted due to concerns over the extent of mucositis present, so discussions regarding parenteral nutrition were being held. Despite four infusions of 20% albumin, her albumin remained severely low, with lowest levels during admission being 12g/L (Table 2). Unfortunately, the patient passed away 10 days after admission secondary to cancer progression. Punch biopsy results were reported post-mortem detailing a vacuolar interface inflammatory reaction consistent with a cutaneous reaction likely erythema multiforme induced by docetaxel (see Appendix). This case draws attention to the possibility of erythema multiforme in patients with mucositis and skin lesions. 

## Discussion

Causality assessment

In SCARs, recognition of the causative agent is extremely important. This is especially pertinent for oncology patients on multi-drug therapies as stopping treatment can affect prognosis detrimentally. In this case, establishing the causative agent was challenging. Docetaxel and Phesgo were administered together during each cycle, meaning either drug could have caused the skin toxicity. There is a singular case report detailing a case of pertuzumab-induced TEN, but no reports of erythema multiforme [11]. Naranjo, a structured scoring system for adverse drug reactions, was used to aid determination of the causative agent [12]. Although both drugs scored within the 'probable' range, the reported literature favoured docetaxel as the most likely cause (Tables 3, 4) [3-7]. A case report described erythema multiforme major with blistering target lesions and mucosal ulceration in a breast cancer patient receiving weekly docetaxel [3]. Moreover, a case series including patients receiving weekly docetaxel therapy reported the highest incidence of developing cutaneous reactions at cycle five, which is similar to our case [6]. Expert opinion from dermatologists and oncologists also attributed the reaction to docetaxel therapy. Therefore, the patient was planned to restart Phesgo after recovery and discontinue docetaxel permanently. This case highlights the difficulty in investigating causative agents in combination therapy and the importance of ongoing pharmacovigilance for all anti-cancer drugs.

**Table 3:** Naranjo Assessment for Adverse Drug Reactions for Docetaxel Docetaxel scores +5 which is ‘probable’ for causing the drug reaction [12].

Question	Yes	No	Do Not Know	Score
1. Are there previous conclusive reports on this reaction?	+1	0	0	+1
2. Did the adverse event appear after the suspected drug was administered?	+2	-1	0	+2
3. Did the adverse reaction improve when the drug was discontinued or a specific antagonist was administered?	+1	0	0	+1
4. Did the adverse event reappear when the drug was re-administered?	+2	-1	0	0
5. Are there alternative causes (other than the drug) that could on their own have caused the reaction?	-1	+2	0	-1
6. Did the reaction reappear when a placebo was given?	-1	+1	0	0
7. Was the drug detected in blood (or other fluids) in concentrations known to be toxic?	+1	0	0	0
8. Was the reaction more severe when the dose was increased or less severe when the dose was decreased?	+1	0	0	0
9. Did the patient have a similar reaction to the same or similar drugs in any previous exposure?	+1	0	0	+1
10. Was the adverse event confirmed by any objective evidence?	+1	0	0	+1
Total Score				+5

**Table 4:** Naranjo Assessment for Adverse Drug Reactions for Phesgo Phesgo scores +4 which is ‘probable’ for causing the drug reaction [12].

Question	Yes	No	Do Not Know	Score
1. Are there previous conclusive reports on this reaction?	+1	0	0	0
2. Did the adverse event appear after the suspected drug was administered?	+2	-1	0	+2
3. Did the adverse reaction improve when the drug was discontinued or a specific antagonist was administered?	+1	0	0	+1
4. Did the adverse event reappear when the drug was re-administered?	+2	-1	0	0
5. Are there alternative causes (other than the drug) that could on their own have caused the reaction?	-1	+2	0	-1
6. Did the reaction reappear when a placebo was given?	-1	+1	0	0
7. Was the drug detected in blood (or other fluids) in concentrations known to be toxic?	+1	0	0	0
8. Was the reaction more severe when the dose was increased or less severe when the dose was decreased?	+1	0	0	0
9. Did the patient have a similar reaction to the same or similar drugs in any previous exposure?	+1	0	0	+1
10. Was the adverse event confirmed by any objective evidence?	+1	0	0	+1
Total Score				+4

Pharmacokinetics of docetaxel

Given that the cutaneous reaction was attributed to docetaxel, understanding the pharmacokinetics is vital. Docetaxel exhibits extensive protein binding predominantly to AAG, an acute phase protein and secondarily to albumin and lipoproteins. Reduced albumin levels can therefore increase the percentage of unbound docetaxel in the bloodstream, which is pharmacologically active [8]. Pharmacokinetic studies have demonstrated that even when total docetaxel concentration is within normal range, increased unbound docetaxel can predict severe toxicities specifically neutropenia [13].

In our patient, prior to cycle two infusion, her albumin levels were 24g/L where she had a grade two skin reaction (Table 2). Prior to cycle five infusion, her albumin levels were moderately low at 23g/L which then deteriorated into severe hypoalbuminemia of 17g/L (Table 2) - at these levels it can be hypothesised that unbound docetaxel levels were raised, increasing toxicity risk. Although we cannot prove causality with a single case report, it is possible that elevated unbound docetaxel exposure predisposes to severe cutaneous reactions. Several studies suggest that AAG is the main factor determining the quantity of unbound docetaxel levels in the blood and therefore is a stronger predictor of toxicities [8,14]. While this is likely the case, AAG is not routinely measured in clinical practice limiting the applicability of these findings. Albumin testing, however, is easily available and inexpensive, making it a useful surrogate marker for reduced protein binding. 

Our patient had a dose reduction of docetaxel, first to 90% in cycle two and then 75% of the standard dose thereafter, yet still developed severe toxicity (Table 1). This raises important questions in regards to dose adjustments in hypoalbuminemic patients. In this case, a dose adjustment to 75% was not successful at mitigating toxicities. Moreover, the patient presented with a milder cutaneous reaction in cycle two which progressed in cycle five despite dose reduction, suggesting cumulative drug exposure may have played a role in developing toxicities. Prospective cohort studies are warranted to determine whether incorporating albumin levels into dosing algorithms can improve the safety of docetaxel usage and other highly protein-bound drugs. 

Hypoalbuminemia and malnutrition

Hypoalbuminemia in patients with cancer is usually multifactorial, secondary to reduced intake, tumour burden and mucositis [8]. In our patient, poor oral intake secondary to mucositis and advanced disease likely contributed to her malnutrition and low albumin levels. There have been studies reporting a statistically significant correlation between hypoalbuminemia and malnourishment with chemotherapy-induced toxicities [15,16]. Nutritional status also affects chemotherapy dosage with malnourished patients more likely to have dose reductions or treatment cessation [9]. Early dietician input with nutritional support and consideration for parenteral nutrition is essential for oncology patients at greater risk of hypoalbuminemia, especially those receiving protein-bound systemic anti-cancer treatment.

Limitations

This case report has several important limitations. As it describes a single case, we cannot determine causality or establish dosing thresholds for albumin levels. The patient's treatment course was likely influenced by multiple factors including sepsis and advancing malignancy which complicates interpretation of findings. Moreover, AAG, the main protein binding to docetaxel, was not measured, hindering full evaluation of this hypothesis. Overall, this case is exploratory and hypothesis-generating, highlighting the need for larger, controlled studies to investigate the observations made. 

## Conclusions

This case highlights a rare but potentially significant link between hypoalbuminemia and docetaxel-induced erythema multiforme. While there have been other case reports for docetaxel-induced SCARs, this correlation has not been previously explored. Our case therefore contributes novel information by suggesting that reduced albumin levels may increase the unbound fraction of docetaxel, potentially amplifying cutaneous toxicity. Clinicians may consider monitoring albumin prior to administration of highly protein bound chemotherapy agents. Early dietician input may help prevent malnutrition reducing the risk of chemotherapy toxicities and need for treatment cessation. As this is a single case report causality cannot be determined therefore further research in this area is needed. Pharmacovigilance studies could help evaluate dosing protocols for taxane therapies incorporating albumin levels and nutritional status to improve patient safety.

## Figures and Tables

**Figure 3:**
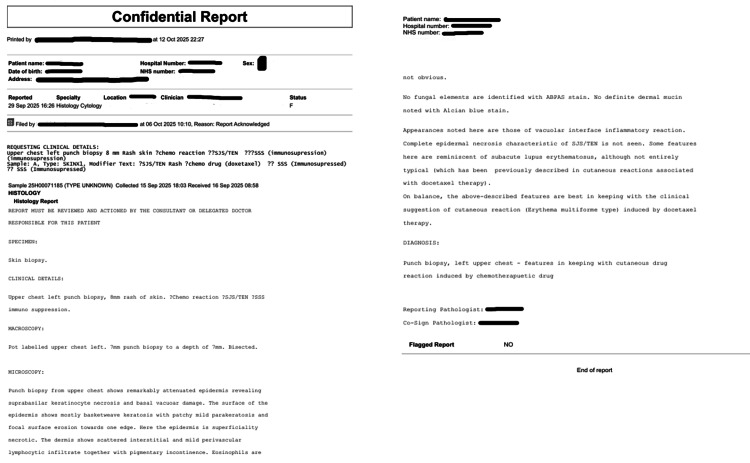
Biopsy report confirming erythema multiforme–type reaction Histopathology report from punch needle biopsy of the upper chest showing features consistent with erythema multiforme type reaction secondary to docetaxel usage.
